# Different Intensities of Exercise and Cardiovascular Performance: A Review: Different Intensities of Exercise and pCardiovascular Performance

**DOI:** 10.31661/gmj.v14i.3722

**Published:** 2025-09-15

**Authors:** Mohsen Davoodi, Foroozandeh Zaravar

**Affiliations:** ^1^ Department of General Courses, School of Paramedical Sciences, Shiraz University of Medical Sciences, Shiraz, Iran

**Keywords:** Cardiovascular Diseases, Cardiomyocytes, Atherosclerosis, Aerobic Exercise, Resistance Training, High Intensity Interval Training [HIIT], Moderate Intensity Continuous Training [MICT]

## Abstract

Cardiovascular diseases [CVDs] remain a major global health concern, with arterial stiffness and atherosclerosis contributing significantly to their prevalence, especially with aging. Regular physical activity is crucial for prevention, improving endothelial function and lipid profiles. This paper examines the effects of different exercise modalities including aerobic training, Moderate-Intensity Continuous Training [MICT], High Intensity Interval Training [HIIT], resistance training [RT] and on cardiovascular health. Aerobic exercise consistently benefits blood pressure, lipid metabolism, and mitochondrial density. HIIT, in particular, often surpasses MICT in enhancing peak oxygen uptake [VO2 peak] and endothelial function. Resistance training improves muscle strength, blood pressure, and insulin sensitivity. However, its impact on arterial stiffness is debated with low to moderate intensity RT appears beneficial, while high intensity RT shows mixed or potentially detrimental effects. Inconsistencies across studies are largely attributed to variations in protocols, intensities, and participant characteristics. Ultimately, exercise is a vital CVD management strategy, underscoring the need for personalized prescriptions based on specific exercise types and intensities to optimize cardiovascular benefits.

## Introduction

Cardiovascular diseases [CVDs] are a major global health concern, standing as a leading cause of death worldwide [1]. Among these, ischemic heart diseases and heart failures are particularly prominent contributors to mortality [2].

Several factors have been linked to the rise of CVDs, including a sedentary lifestyle, diabetes, stress, declining physical function, dental caries, and periodontal diseases [1][3]. Given the global increase in the elderly population and extended life expectancies due to technological and medical advancements, caring for older adults has become a significant public health challenge [4]. Global life expectancy at birth has seen a significant increase, rising from 64.2 years in 1990 to 72.6 years in 2019 [5]. This positive trend is projected to continue, with an estimated average life expectancy of 77.1 years worldwide by 2050 [5].

The cardiovascular system undergoes subtle physiological changes with aging [6]. Atherosclerosis [hardening of the arteries] is the key factor in many cardiovascular diseases [7]. It is a chronic inflammatory disease marked by abnormal lipid metabolism, leading to the accumulation of lipids in large arteries, with subsequent calcification occurring within these plaques [8]. It shares similarities with autoimmune diseases due to immune responses against self-components in the arterial wall [9].

Autoreactive T cells recognize oxidized low-density lipoproteins [oxLDL], triggering inflammation and plaque formation. These lipid-rich plaques, found in the arterial intima, contain a mix of immune, non-immune, and apoptotic cells, contributing to cardiovascular complications and global mortality [10].

Cholesterol accumulates in macrophage cells when they cannot effectively remove it, leading to the formation of foam cells. These foam cells, which are swollen macrophages filled with low-density lipoprotein [LDL], contribute to physical obstruction in blood vessels [11].

Numerous studies have examined the impact of physical activities in promoting positive cardiovascular adaptations, reducing LDL, and increasing HDL [12].

In fact, the lack of physical activity is one of the most significant modifiable risk factors for CVD [13]. The primary effects of physical activity on the vascular endothelium are realized through increased shear stress on the vessel walls, which enhances endothelial function and promotes vasodilation, ultimately improving cardiac output during physical activity [14].

The direct effects of aerobic exercise can lead to increased calcium influx, enhanced PI3K activity, reduced tissue fibrosis, and decreased apoptosis in cardiomyocytes [15]. Additionally, numerous studies have explored how different exercise intensities can induce angiogenesis in hypoxic tissues [16].

Panjepour et al. investigated the role of adenosine and its receptors in promoting angiogenesis in hypoxic tissues and inducing vessel formation under hypoxic conditions [17].

This correlates with research showing that regular physical activity, mechanical stress on the vascular wall, adenosine, and hypoxia increase vascular endothelial growth factor [VEGF], leading to angiogenesis in cardiomyocytes [16][18].

## Vascular Changes and Atherosclerosis

With physiological aging, the thickness and stiffness of vascular walls progressively increases [19]. This phenomenon is primarily driven by several interconnected structural alterations within the vessel wall [20]. Key contributions include the accumulation and disorganization of collagen fibers, a major component of the extracellular matrix, alongside the fragmentation and degradation of elastic lamellae [elastin fibers] of blood vessels [19]. Furthermore, these changes are frequently exacerbated by the deposition of calcium salts [calcification], which contributes significantly to the loss of arterial compliance and elasticity [19]. These combined processes lead to a stiffer, less compliant vasculature, with profound implications for cardiovascular function [21]. As a result of this decreased vascular compliance, the arterial system loses its ability to effectively buffer the pulsatile flow from the heart, leading to an increased pulse pressure and an accelerated pulse wave velocity [22]. This condition makes elderly individuals susceptible to isolated systolic hypertension, which is the most common type of hypertension in older adults [23]. The increase in intima-media thickness of blood vessel walls is an important marker of atherosclerosis and has predictive value for the occurrence of cardiovascular events [24]. A study by Fakhrzadeh and colleagues showed that the intima-media thickness of the carotid artery in women with a history of gestational diabetes is greater compared to women without such a history [25].

In most studies, regular physical activity has been suggested as a major preventive factor [26][27]. Several factors have been identified in the etiology and investigation of atherosclerosis, encompassing both lifestyle and genetic influences [28]. In addition to metabolic abnormalities related to fats and lipoproteins, high plasma cholesterol levels, especially low-density lipoprotein [LDL] cholesterol contribute to the progression of atherosclerosis [29].

High-density lipoproteins [HDL] perform both antioxidant and anti-inflammatory activities [30]. Additionally, HDL is recognized for its effective role in cardiovascular protection through reverse cholesterol transport [31]. Patients with low HDL levels are primarily susceptible to an increased risk of coronary atherosclerosis [32]. Numerous studies have shown a relationship between fasting triglyceride [TG] levels and cardiovascular diseases; however, through multivariate analysis of variance, TG tends to be recognized as an independent risk factor in cardiovascular diseases compared to HDL cholesterol [33][34]. An inverse relationship between physical fitness and mortality from cardiovascular diseases has been reported [35]; furthermore, increased levels of physical activity and fitness are associated with a reduction in cardiovascular diseases [36].

Among patients with a history of stable cardiovascular diseases, the mortality rate is lower than that of those who do not participate in exercise programs [37][38]. Regular exercise increases plasma NOx [nitrite/nitrate] levels in middle-aged and elderly individuals [39], leading to improvements in hypertension, increased blood flow, and vascular flexibility [40][41][42]; thus, by affecting plasma adiponectin and NOx, it prevents the onset and progression of cardiovascular disorders in type 2 diabetic patients [43][44]. In summary, the metabolism of fats, lipoproteins, and endothelial dysfunction are major factors in the development of atherosclerosis; while studies indicate the impact of exercise on lipoprotein metabolism and endothelial function of blood vessels.

## Factors Contributing to Cardiovascular Diseases in the Elderly

The aging process is a physiological phenomenon that, if metabolic risk factors are not controlled, can lead to a pathological process, gradually resulting in the death of individuals [45]. During the aging process, the heart muscle becomes stiff due to the increased size of the ventricles, and there is an increase in the extracellular matrix and collagen, leading to myocardial fibrosis [46]. These structural changes gradually result in an increase in the left ventricular [LV] wall thickness [46]. Left ventricular hypertrophy [LVH], heart failure [particularly heart failure with preserved ejection fraction], and atrial fibrillation are common cardiovascular conditions that increase significantly in prevalence with age, even in elderly individuals without a primary diagnosis of concurrent hypertension or other overt cardiovascular disease [47][48][49][50][51]. LVH is independently associated with an increased risk of coronary heart disease [CHD], sudden cardiac death [SCD], and strokes [52][53][54][55]. The systolic function of the left ventricle, measured by the LV ejection fraction, remains relatively preserved in older age , however, the maximum end-diastolic volume [EDV], which is considered a positive adaptation to continuous exercise, decreases in non-diseased and non-exercising elderly individuals [56][57][58][59]. Furthermore, in older adults, the cardiac muscle's response to β-adrenergic stimulation diminishes, while it tends to increase during physical activities, resulting in a decreased reserve of the heart's contractile strength [6][60][61]. Furthermore, the maximum heart rate during regular physical activities declines in individuals over the years, which is the main reason for the decrease in cardiac output reserve during exercise in the elderly [62][63].

The variability of heart rate decreases steadily and consistently with age, due to the disruption of the autonomic control system of the heart as one age, and is an indicator of the risk of mortality in later years [64]. Silent heart attacks and valvular heart diseases, aortic valve sclerosis, and other valve-related problems occur most frequently in the elderly [65]. As age increases, the concentration of cholesterol gradually rises in both men and women, but after the fifth decade of life, this concentration stabilizes in men while the trend continues to rise in women [66][67][68].

## Exploring the Impact of Different Types and Intensities of Exercise in Patients with Cardiovascular Conditions

### Aerobic Exercise

According to The American College of Sports Medicine [ACSM], aerobic exercise is any rhythmic activity that engages large muscle groups and can be sustained over an extended period of time [69]. Some research indicates that aerobic exercise primarily enhances cardiovascular fitness and improves lipid profiles [70]. A recent systematic review by Mousavi Zadeh et al. underscores the beneficial effects of aerobic exercise on cardiovascular health in individuals with type 2 diabetes. The results demonstrate that participation in aerobic activities can significantly lower both systolic and diastolic blood pressure [SBP/DBP], primarily by enhancing nitric oxide [NO] production, which reduces arterial stiffness. Furthermore, aerobic exercise supports lipid metabolism by increasing lipoprotein lipase activity, which helps regulate lipid balance, by enhancing the HDL-to-LDL ratio, and increases mitochondrial density. Additionally, it positively affects autonomic balance and reduces sympathetic nervous system activity, contributing to better blood pressure regulation [70].

In 2002, Wisløff and his team demonstrated the beneficial effects of aerobic exercise on the myocardium of rats following an ischemic event [71]. Building on that research, Wisløff and his team found similar outcomes in 2007 involving 27 patients with stable post-infarction heart failure, participants were randomized to either moderate continuous training [70% of peak heart rate] or aerobic interval training [95% of peak heart rate], with a control group receiving standard physical activity advice. The aerobic interval training group, which exercised at a higher intensity, showed significantly greater improvements in peak oxygen uptake [VO2peak] compared to the moderate continuous training group. Additionally, only the aerobic interval training led to reverse LV remodeling, with reductions in both LV end-diastolic and end-systolic volumes, as well as an increase in LV ejection fraction.

Improvements in endothelial function, as measured by brachial artery flow-mediated dilation, were also more pronounced in the aerobic interval training group. Furthermore, mitochondrial function in the lateral vastus muscle improved exclusively with aerobic interval training. Both exercise groups reported enhancements in quality of life, while no changes were observed in the control group [72]. This paper illustrates that exercise intensity is vital for reversing LV remodeling and improving aerobic capacity, endothelial function, and quality of life in patients with post-infarction heart failure [72].

### Resistance Training [RT]

Resistance training is a type of exercise that evokes muscular contraction against an external force [73].

Some studies show that resistance training not only enhances or maintains muscle mass and strength but also offers positive physiological and clinical benefits for CVD and its associated risk factors [73][74][75][76].

Mousavi Zadeh and his colleagues reviewed studies investigating resistance training interventions, lasting from 8 to 52 weeks, consistently reveal positive effects on important risk factors for CVD [70]. They demonstrate that RT has been found to significantly lower both SBP and DBP and also has a positive impact on lipid profiles, leading to increase in HDL cholesterol and modest decreases in triglyceride levels, which together improve overall lipid metabolism [70]. Participants in resistance training programs experienced an average increase of 2 kg in lean muscle mass, which directly enhances glucose uptake in muscle tissue and boosts insulin sensitivity. Improved insulin sensitivity is crucial for managing hyperglycemia and lowering cardiovascular risk by reducing oxidative stress and inflammation [70]. Additionally, resistance training has been associated with an anti-inflammatory effect, evidenced by an average 5% decrease in C-reactive protein [CRP] levels across various studies, along with variable changes in IL-6 [70]. These results underscore the effectiveness of resistance training as a valuable strategy for reducing the risk of cardiovascular disease and potentially slowing the progression of atherosclerosis, particularly in those with type 2 diabetes and have been confirmed by other studies [77]. In contrast, some others find no effect in young and healthy subjects, persons at risk for cardiovascular disease, and those with metabolic syndrome [78][79][80][81]. Additionally, some research indicates that RT may actually increase arterial stiffness in healthy young individuals, leading to decreased vascular compliance [82][83]. The impact of RT on arterial stiffness remains debated [84] although the results of recent studies are more in favor of positive effects for some populations [70][77].

In 2021, Zhang et al. studied the effects of different intensities of resistance training [RT] on arterial stiffness in young and middle-aged adults. While the meta-analysis did not find a significant impact on pulse wave velocity [PWV], a marker of arterial stiffness, a meta-regression analysis indicated a correlation between RT intensity and PWV. Their subgroup analysis revealed that low to moderate intensity RT effectively reduces PWV in young and middle-aged adults, suggesting its potential in reversing arterial stiffness. This is while high-intensity RT did not effectively reduce pulse wave velocity [PWV] in either age group [84]. Their findings are in contrast with similar previous works such as Miyachi et al. and Ashor et al. [85][86]. There are also findings which indicate an increase in arterial stiffness after high intensity RT in young subjects [86]. Another study indicates that moderate intensity resistance exercise [60%, 1-RM] in middle-aged women did not adversely affect vascular health and resulted in increased muscle strength [87]. These results suggest that the effects of low to high intensity RT on arterial stiffness are not yet fully understood but there is a deference between intensities of RT regarding arterial stiffness [84][88]. regardless of intensity, Figueroa et al. suggested that overall resistance exercise can reduce central and peripheral blood pressure in middle-aged and older adults with elevated baseline blood pressure [88]. Nevertheless, the inconsistencies in findings may be due to variations in study criteria, RT protocols and intensities, and participant characteristics [84].

## Comparative Effects of High-Intensity Interval Training [HIIT] and Moderate-Intensity Continuous Training [MICT] on Cardiovascular Health

HIIT and MICT are two widely utilized exercise modalities, yet their relative effectiveness in enhancing cardiovascular fitness among patients with coronary artery disease [CAD] remains a topic of ongoing research [89]. HIIT is characterized by short bursts of intense activity followed by periods of rest or lower intensity [89]. This approach has gained popularity for its potential to improve aerobic capacity and reduce cardiovascular risk factors in a shorter time frame. Conversely, MICT, which involves sustained moderate-intensity exercise over longer durations, has long been the foundation of cardiac rehabilitation programs [90]. As research continues to explore the nuances of these modalities, understanding their comparative effectiveness will be crucial for optimizing exercise prescriptions in this population.

As explained before, arterial stiffness is a significant indicator of future cardiovascular events and overall mortality [91]. There is much evidence that shows the positive effects of aerobic exercise on arterial stiffness [85]. While Oliveira et al. noted that this effect may not be present in obese and overweight men, there is insufficient evidence to draw conclusions about women [92]. They have conducted a study which investigated the effects of 8 weeks of HIIT and MICT on arterial stiffness and central blood pressure [BP] in young obese women where both HIIT and MICT significantly reduced carotid-femoral pulse wave velocity [CFPWV], a measure of arterial stiffness [92]. Also, MICT showed significant reductions in brachial and central DBP [92]. They suggest that the reduction in CFPWV after aerobic training could be directly related to the reduction in elastin breakdown and collagen deposition on arterial layer and Indirectly, it might be related to improvements in oxidative stress and inflammation, as well as reductions in sympathetic nervous activity [SNA] [92].

They suggest that HIIT is superior to MICT in improving endothelial function [92]. This paper also touches upon why their findings regarding Augmentation Index [AIx] responses differ from some other studies, particularly noting that women may experience a greater reduction in AIx than men [92]. This may be attributed to women having greater sensitivity to β-adrenergic receptors, which can counteract α-adrenergic vasoconstriction, resulting in less vasoconstriction for a given level of sympathetic nerve activity [SNA]. Additionally, this could be linked to the direct effects of estrogen in enhancing β-adrenergic receptor sensitivity or indirectly through increased availability of nitric oxide [NO], which may promote β-adrenergic-mediated vasodilation in the peripheral vasculature [92]. In addition to their direct effects on arterial health, research also evaluates HIIT and MICT in relation to other significant health outcomes. A recent meta-analysis of randomized controlled trials involving cancer survivors revealed that while HIIT was significantly more effective than MICT in enhancing peak oxygen uptake [VO2 peak], a crucial measure of cardiopulmonary function, no statistically significant differences were found between HIIT and MICT in terms of improvements in body composition or physical function [93]. This indicates that although HIIT may provide greater benefits for cardiopulmonary fitness, both training modalities can produce similar outcomes in other important areas, such as body composition and overall physical function in specific populations [93]. Another study by Tahir and his colleges showed similar results in patients with Coronary Artery Disease [CAD] [89].

Their results demonstrate that HIIT significantly surpassed MICT in enhancing resting heart rate and VO2 max, a crucial measure of cardiovascular fitness, while also indicating no significant difference in SBP and DBP between the two groups, despite observing a reduction in both. They also suggest that while both exercise modalities are effective in managing blood pressure in patients with coronary artery disease [CAD], HIIT offers additional advantages in enhancing overall cardiovascular fitness [89]. Also, Shorter durations of HIIT may be a suitable alternative for individuals with limited cardiac output during exercise [94].

Compared to MICT, HIIT seems to provide greater advantages for cardiovascular fitness, potentially having a more pronounced effect on women. However, to validate these findings and evaluate the long-term sustainability of HIIT's benefits, future research should include larger sample sizes, longer follow-up periods, and a focus on gender differences to better understand how HIIT may affect men and women differently [89][92][95].

### Concurrent Trainings

Concurrent training [CT] integrates both endurance and strength exercises into a single workout routine, harnessing the advantages of each form of training [96]. This method has been shown to lead to notable improvements in glycemic control, body composition, cardiorespiratory fitness [CRF], endothelial function, artery stiffness lipid profiles, inflammatory markers, and insulin sensitivity [97][98]. Additionally, the synergistic effects of concurrent training may enhance cardiac autonomic function and reduce the risk of cardiac autonomic neuropathy [CAN], addressing critical issues related to type 2 diabetes mellitus [T2DM] and its complications [97]. Zaki et al. demonstrated that CT significantly improves cardiac autonomic modulation, metabolic profiles, body composition, CRF, and overall quality of life in individuals with T2DM and CAN [96].

In 2022, Khalafi et al. found that concurrent training [CT] was significantly more effective than resistance training [RT] at increasing cardiorespiratory fitness [CRF], as measured by VO2max/peak. Additionally, CT outperformed aerobic training [AT] in enhancing both lower-body and upper-body muscular strength. However, there were no significant differences in CRF improvements between CT and AT, nor in muscular strength gains [both lower and upper body] between CT and RT. The study concluded that CT is an effective strategy for improving both CRF and muscular strength in middle-aged to older adults, without negatively affecting the benefits derived from either AT or RT alone, indicating that CT is a viable and beneficial exercise approach for promoting healthy aging [99].

In 2022, Bouamra et al. found that concurrent training [CT] was more effective than both high-intensity interval training [HIIT] and resistance training [RT] alone for improving body composition, resulting in greater fat loss and reductions in body mass, as well as enhancing cardiorespiratory fitness, as measured by the 6-minute walking test distance and VO2max. Additionally, CT demonstrated greater improvements than HIIT in the medicine ball throw test, which assesses upper body power.

However, RT alone outperformed both HIIT and CT in enhancing relative handgrip strength and countermovement jump [CMJ] performance, indicators of lower body power. The study concluded that this specific CT program, characterized by a defined intensity and volume [50% RT and 50% HIIT], serves as an effective exercise intervention for addressing obesity in youth, leading to significant improvements in body composition and cardiorespiratory fitness. While RT showed superiority in certain strength measures, CT provided broader benefits for fat loss and overall fitness [100]. Overall, concurrent training [CT] is an exceptionally effective strategy for enhancing cardiorespiratory fitness, muscular strength, and body composition in various populations, ranging from middle-aged adults to obese youth, often yielding greater benefits than either training type alone. This integrated approach is recognized as a viable and advantageous exercise method for improving overall health and well-being [96][99][100]. In 2024, Baghdadabadi et al. conducted a study examining the effects of an 8-week concurrent training program, which included both aerobic and resistance training, on blood and vascular biomechanics in 60 male and female patients with coronary artery disease who had undergone angioplasty.

The findings revealed that concurrent training significantly improved the resting lumen diameter of the left femoral artery during both systolic and diastolic phases, with these enhancements being consistent across genders, indicating no significant differences in the training's effectiveness for these vascular measures. Although the training yielded overall positive results, there were no significant changes observed in artery compliance, blood flow intensity, intima-media thickness ratio, blood flow velocity, or blood pressure. In conclusion, the study advocates for concurrent training at appropriate intensity and duration for middle-aged men and women following coronary artery angioplasty to improve blood and vascular biomechanics [101]. In 2011, Mosti et al. investigated the effects of an 8-week concurrent training program that combined maximal RT and plantar flexion [PF] endurance training on walking ability in 10 patients with peripheral arterial disease [PAD].

The findings revealed that the training group experienced significant improvements in treadmill peak oxygen consumption and time to exhaustion, both exceeding 12%. Additionally, they achieved substantial gains in leg press maximal strength [38.3%] and rate of force development [140.1%], along with a modest enhancement in work economy. The study concluded that this concurrent training approach yields positive training responses in PAD patients comparable to those from RT or plantar flexion [PF] endurance training alone, with no reported adverse effects. This indicates that combining these exercise modalities is a safe and effective method for enhancing walking ability in individuals with PAD [102]. The positive effects of concurrent training have also been observed in various outcomes and populations, including patients with chronic heart failure [103], coronary artery disease [104][105] and those with hypertrophic cardiomyopathy [106].

In conclusion, concurrent training is a highly effective and versatile exercise strategy, consistently showing broad benefits for cardiorespiratory fitness, muscular strength, body composition, and vascular health across diverse populations and both genders [105]. It often provides comprehensive improvements that are comparable or superior to single-modality training. Although many studies reported no adverse effect from CT, there is insufficient data on concurrent training [CT] to draw definitive conclusions [104].

## Discussion

An inverse relationship has been reported between physical fitness and mortality due to CVD [35]. Additionally, increased levels of physical activity and fitness are associated with a reduction in CVD [36]. Among patients with stable cardiovascular diseases, the mortality rate is lower for those who participate in exercise programs compared to those who do not [107][108]. Rajar et al. demonstrate that high-intensity interval training [HIIT] serves as an efficient and effective exercise option for young, sedentary, and inactive men. Their study supports the notion that HIIT can be utilized as a non-pharmacological strategy to enhance physical health, improve fitness levels, and lower the risk of cardiovascular diseases [109]. HIIT provides a time-efficient and cost-effective alternative for individuals with limited workout time who still seek desirable health and fitness outcomes [109]. A lengthy period of endurance training has a notable impact on stroke volume and the autonomic regulation of the heart [110]. This type of training leads to increased parasympathetic activity and decreased sympathetic activity in the resting heart of individuals, resulting in a lower intrinsic heart rate during rest due to these exercise-induced changes [110]. Studies have indicated that a season of moderate-intensity exercise enhances endothelial function and mitigates the adverse effects of high-fat diets in both lean and obese individuals. However, these benefits do not extend to patients with type 2 diabetes [110][111][112].

In 1981, Magder and colleagues investigated the effects of swimming and cycling exercises performed on a water ergometer at submaximal intensity in patients recovering from a heart attack. Their findings indicated that these exercises modified oxygen consumption conditions and enhanced blood flow to the cardiac tissues of the patients. The buoyancy and horizontal positioning of the patients in the water increased central blood volume, which, with repeated sessions, could lead to heightened peripheral vascular resistance compared to standing.

The compensatory effects of water on the body, along with reduced cutaneous blood flow, body positioning during swimming, and physical activity, collectively contribute to increased ventricular afterload, improved oxygen uptake, and enhanced cardiac output in these patients [107].

Additionally, Gielen and colleagues [2010] discovered that endurance exercises in patients with systolic heart failure can enhance both systolic and diastolic function of the left ventricle while reducing diastolic diameter in these individuals [27]. The findings of Rezai and Davoudi [2022] demonstrated that regular physical activity, when combined with the herbal antioxidant and anti-inflammatory agent curcumin, can significantly help mitigate cardiovascular disorders and injuries, potentially facilitating the repair of myocardial cell damage [113].

Regular physical activity, while maintaining normal endothelial cell function and NO release, prevents atherosclerosis and improves endothelial cell function in patients with coronary artery disease [13][114][115]. Among various types of exercises, aerobic exercises contribute to greater exercise performance and promote a healthier cardiovascular system [116][117]. Studies also show that moderate-intensity aerobic exercises and short-term resistance exercises can modulate blood pressure responses in hypertensive patients [118][119][120]. On the other hand, endurance training strengthens the cardiovascular system, leading to significant changes in metabolism and lipid processes, including meaningful reductions in weight, body mass, and especially fat percentage and adipose tissue [as a major producer of inflammatory cytokines] [121].

Additionally, there are reports indicating that the improvements are associated with the volume of activity rather than the intensity of exercise or enhancements in fitness [122]. Concerning exercise types, many studies indicate that combining aerobic training with resistance training may provide greater benefits for cardiovascular disease risk factors and indicators compared to engaging in either type of exercise alone [123][124][125][126].

Regarding concurrent training, it is an effective and versatile exercise strategy that consistently enhances cardiorespiratory fitness, muscular strength, body composition, and vascular health across various populations and genders. It often yields comprehensive improvements that are comparable to or better than single-modality training [103][104][106][126].

Finally, considering the necessity of pharmacological interventions prescribed for cardiac patients, recent studies by Davoodi and colleagues have demonstrated that a combination of 12 weeks of regular exercise and the use of cholesterol-lowering medications such as atorvastatin significantly enhances the reduction of hypercholesterolemia, lowers blood pressure, and increases blood flow in patients with coronary heart disease [127]. This mechanism is achieved through the reduction of apolipoproteins [Apo_A1], C-reactive proteins, and atrial natriuretic peptides [ANP], ultimately leading to the attenuation of cardiovascular diseases and improvement in the structure and function of this group of patients [127].

In general, regular physical activity can prevent the occurrence of cardiovascular diseases and improve the quality of life for patients with chronic heart failure, as well as assist in the treatment of certain types of these conditions [128][129]. Practical recommendations for patients with pulmonary heart disease include participating in concurrent or submaximal aerobic exercise alongside low-intensity resistance training or HIIT [for individuals with limited cardiac output]; however, this should be done with caution and under strict medical supervision tailored to each patient's condition.

## Conflict of Interest

None to declare.
